# Increasing surgical rate of clavicle fractures and acromioclavicular dislocations in Chile: analysis over the last 15 years reveals disparities in access according to insurance type

**DOI:** 10.1186/s12891-024-07769-4

**Published:** 2025-04-02

**Authors:** Catalina Vidal, Rodrigo de Marinis, Rodrigo Liendo, Isadora Silva, María Jesús Lira, Julio J Contreras

**Affiliations:** 1 Department of Orthopedics and Trauma, Pontificia Universidad Católica de Chile, Diagonal Paraguay #362, Santiago, 8330077 Chile; 2 Shoulder and Elbow Unit, Complejo Asistencial Dr. Sótero del Río, Santiago, Chile

**Keywords:** Clavicle fracture, Acromioclavicular joint dislocation, Surgery, Insurance

## Abstract

**Background:**

In recent years, an increase in surgeries to treat clavicle injuries has been reported. It has been hypothesized that the studies regarding the beneficial effect of surgery in patients with displaced clavicle fractures may have contributed to raise the surgical rates for injuries around the clavicle. To our knowledge, there is a lack of data from Latin American countries on surgical rates of clavicle-related surgeries. The aim of this study is to describe the rate of clavicle surgeries, including clavicle fracture and acromioclavicular dislocation, in the last 15 years and to analyze the possible effect of sex, age, and health insurance in those rates.

**Methods:**

An observational cross-sectional study was carried out. Patients over 18 years old diagnosed with the following ICD-10 codes were selected: S420 "Clavicle fracture", S431 "Dislocation of the acromioclavicular joint", and S435 "Sprains and strains of the acromioclavicular joint". We collected information on the year of surgery, sex, age and type of insurance. The annual rate of surgeries and the rate for the period studied per 100,000 people were calculated. The rate was compared through negative binomial regression, reporting Incidence Rate Ratios (IRR) with 95% confidence interval (95% CI).

**Results:**

During a 15 years period of observation, 24,570 surgeries were performed. For clavicle fractures an 8.0 × 100,000 surgical rate was observed, and a 4.7 × 100,000 rate was found for acromioclavicular dislocations. The surgical rate for clavicular injuries increased from 2.8 in 2005 to 19.1 in 2019. Rates were higher in men, and ages between 20 and 35 years. The surgical rate for clavicular injuries in the public system was 11.1 × 100,000 and 30.9 × 100,000 in the private system, which represents a difference of 2.8 times between those healthcare systems.

**Conclusion:**

There has been a significant increase in clavicle and acromioclavicular dislocation surgeries in Chile, with disparities influenced by age, gender, and type of health insurance.

## Introduction

Clavicle fractures are frequent, especially among active individuals and athletes [1]. Though acromioclavicular dislocations are not as frequent, both conditions can cause significant functional impairment. Shoulder girdle injuries usually occur as a result of high-energy trauma or sports-related injuries, such as, falling onto the shoulder, a direct blow to the clavicle, or overuse injuries due to repetitive stress [2].

Treatment options can include rest, immobilization, physical therapy, or surgery depending on the severity of the injury [3]. In 2007, the Canadian Orthopedic Trauma Society conducted a randomized clinical trial that compared nonoperative management to operative management for displaced clavicle fractures. At one-year follow-up, patients who underwent surgery showed significantly better functional outcomes than those who underwent conservative management [4]. These findings have been replicated over time by different authors and in 2019 a meta-analysis summarized the data on this topic [5].

In recent years, an increase in surgeries to treat clavicle injuries has been reported [6, 7]. It has been hypothesized that the aforementioned studies regarding the beneficial effect of surgery in patients with displaced clavicle fractures may have contributed to raise the surgical rates for injuries around the clavicle [7]. To our knowledge, there is a lack of data from Latin American countries on surgical rates of clavicle-related surgeries. Therefore, the aim of this study is to describe the rate of clavicle surgeries, including clavicle fracture and acromioclavicular dislocation, in the last 15 years and to analyze the possible effect of sex, age, and health insurance in those rates.

### Methods

An observational cross-sectional study was carried out with approval of the Institutional Review Board (ID. 16–196). Data was obtained from the Department of Statistics and information of the Ministry of Health of Chile from 2005 to 2019 (https://deis.minsal.cl). By law, this department systematically and prospectively collects data corresponding to all discharges from the national healthcare system including public and private practice-based institutions. That data is then used to create an open database for research analysis and statistics in health.

Patients over 18 years old diagnosed with the following ICD-10 codes were selected: S420 "Clavicle fracture", S431 "Dislocation of the acromioclavicular joint", and S435 "Sprains and strains of the acromioclavicular joint". We collected information on the year of surgery, sex, age and type of insurance (public or private). In Chile, healthcare is funded through a mandatory 7% deduction from patients´ salaries, allocated to either a public (FONASA) or private health insurance (ISAPRES).

Surgery data were characterized using descriptive analysis. The annual rate of surgeries and the rate for the period studied per 100,000 people were calculated. Data of the population size by year and age range were obtained from the National Statistics Institute (https://www.ine.gob.cl/). The rate was compared through negative binomial regression, reporting Incidence Rate Ratios (IRR) with 95% confidence interval (95% CI). Statistical analyses were performed with Stata v.16 software and significance set at 5%.

### Results

**Fig. 1:**
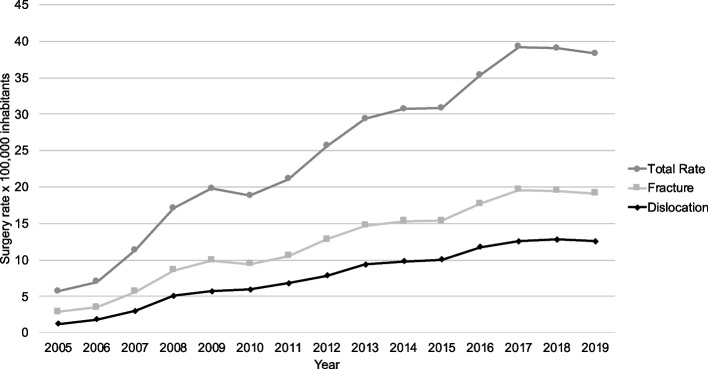
Surgery rate by 100,000 inhabitants of clavicle fracture and dislocation between 2005 and 2019

During a 15 years period of observation, 24,570 surgeries were performed for clavicle fracture and acromioclavicular dislocation in patients over 18 years of age. 86% (21,207) were male patients, and the mean age was 37.4 ± 13.8 years old. The surgery rate for all the included patients considering the entire studied period was 12.96 (12.8–13.1) × 100,000 inhabitants. For clavicle fractures an 8.0 × 100,000 surgical rate was observed, and a 4.7 × 100,000 rate was found for acromioclavicular dislocations. There was a statistically significant difference (IRR 1.7; 95% CI 1.1–2.8) between the two procedures (Fig. 1).

Adjusting by year, a mean annual increase of 15% was observed in surgery rates (95%CI 11%-19%). The surgical rate for clavicular injuries increased from 2.8 in 2005 to 19.1 in 2019, representing a 10.2-fold (95%CI 8.6–12.1) rise over that period (Table 1).

**Table 1:** Overall rate of clavicle surgery, fracture rate and acromioclavicular dislocation

Total surgery	IRR	Std. Err	p-value	IC 95%	
Reference 2005					
2006	1.22	0.09	0.007	1.06	1.42
2007	1.99	0.14	< 0.001	1.74	2.27
2008	3.01	0.19	< 0.001	2.65	3.41
2009	3.48	0.22	< 0.001	3.08	3.93
2010	3.30	0.21	< 0.001	2.92	3.74
2011	3.70	0.23	< 0.001	3.27	4.18
2012	4.50	0.27	< 0.001	3.99	5.07
2013	5.16	0.31	< 0.001	4.59	5.81
2014	5.39	0.32	< 0.001	4.79	6.06
2015	5.41	0.32	< 0.001	4.81	6.09
2016	6.22	0.37	< 0.001	5.53	6.98
2017	6.89	0.41	< 0.001	6.14	7.74
2018	6.85	0.40	< 0.001	6.11	7.69
2019	6.73	0.40	< 0.001	5.99	7.55
**Fracture**	**IRR**	**Std. Err**	**p-value**	**IC 95%**	
Reference 2005					
2006	1.46	0.16	0.001	1.18	1.81
2007	2.41	0.24	< 0.001	1.98	2.93
2008	4.09	0.38	< 0.001	3.40	4.91
2009	4.58	0.42	< 0.001	3.82	5.49
2010	4.79	0.44	< 0.001	4.00	5.74
2011	5.49	0.50	< 0.001	4.60	6.57
2012	6.31	0.57	< 0.001	5.29	7.53
2013	7.55	0.67	< 0.001	6.34	9.00
2014	7.92	0.70	< 0.001	6.66	9.43
2015	8.11	0.72	< 0.001	6.82	9.65
2016	9.50	0.84	< 0.001	7.99	11.29
2017	10.14	0.89	< 0.001	8.53	12.04
2018	10.33	0.91	< 0.001	8.70	12.27
2019	10.16	0.89	< 0.001	8.56	12.07
**Acromioclavicular dislocation**	**IRR**	**Std. Err**	**p-value**	**IC 95%**	
Reference 2005					
2006	1.04	0.11	0.697	0.85	1.27
2007	1.67	0.16	< 0.001	1.39	2.00
2008	2.17	0.19	< 0.001	1.83	2.59
2009	2.63	0.23	< 0.001	2.22	3.12
2010	2.16	0.19	< 0.001	1.82	2.57
2011	2.32	0.20	< 0.001	1.95	2.75
2012	3.10	0.26	< 0.001	2.63	3.66
2013	3.32	0.28	< 0.001	2.82	3.91
2014	3.44	0.28	< 0.001	2.92	4.04
2015	3.34	0.28	< 0.001	2.84	3.92
2016	3.69	0.30	< 0.001	3.14	4.34
2017	4.40	0.35	< 0.001	3.75	5.15
2018	4.18	0.34	< 0.001	3.57	4.90
2019	4.08	0.33	< 0.001	3.48	4.78

**Fig. 2:**
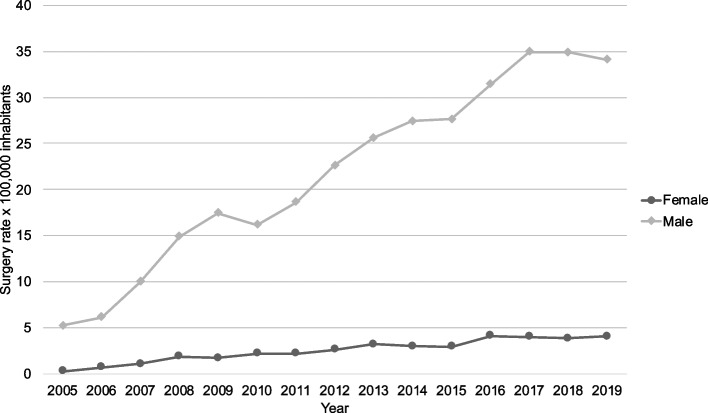
Surgery rate by 100,000 inhabitants between 2005 and 2019 by sex

Rates were higher in men compared to women (22.6 [CI95%22.3–22.9] versus 3.4 [CI95%3.3–3.5] IRR: 8.90 [CI95%8.07–9.11 *p* < 0.001]) (Fig. 2). The rate of clavicle fracture surgery where 13.8 [CI95%13.5 -14.0] in men and 2.61 [CI95%2.5–2.7]; and for acromioclavicular dislocation surgery 8.8 [CI95%8.6–9.0] for men and 0.7 [CI95%0.7–0.8] for woman.

**Fig. 3:**
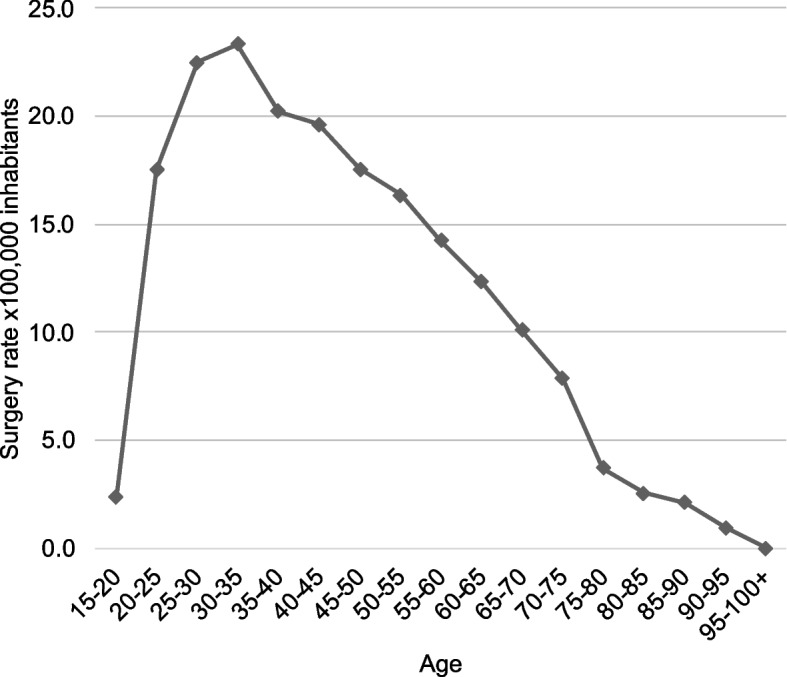
Surgery rate by 100,000 inhabitants of clavicle fracture and dislocation between 2005 and 2019 by age groups

Highest rate was observed in ages between 20 and 35 years (Fig. 3). Also, the greatest overall increase in surgery rate occurred among individuals aged 25 to 34. Analyzed by diagnosis, the 15–24 age group showed a higher increase in surgeries for clavicle fractures, while the 25–34 age group had a greater increase in surgeries for acromioclavicular dislocations (Table 2).

**Table 2:** Surgery rate between 2005 and 2019 according to age group

Total surgery	IRR	Std. Err	p-value	IC 95%	
15–24 (reference)					
25–34	1.06	0.20	0.754	0.74	1.52
35–44	0.92	0.17	0.670	0.64	1.33
45–54	0.76	0.14	0.142	0.53	1.10
55–64	0.55	0.10	0.001	0.38	0.79
65–74	0.32	0.06	< 0.001	0.22	0.47
> 75	0.15	0.03	< 0.001	0.10	0.22
**Fracture**	**IRR**	**Std. Err**	**p-value**	**IC 95%**	
15–24 (reference)					
25–34	0.88	0.19	0.543	0.58	1.33
35–44	0.72	0.15	0.117	0.48	1.09
45–54	0.57	0.12	0.009	0.38	0.87
55–64	0.45	0.09	< 0.001	0.30	0.68
65–74	0.28	0.06	< 0.001	0.19	0.43
> 75	0.14	0.03	< 0.001	0.09	0.22
**Dislocation**	**IRR**	**Std. Err**	**p-value**	**IC 95%**	
15–24 (reference)					
25–34	1.63	0.26	0.002	1.19	2.22
35–44	1.57	0.25	0.004	1.15	2.14
45–54	1.35	0.21	0.058	0.99	1.84
55–64	0.88	0.14	0.445	0.65	1.21
65–74	0.45	0.08	< 0.001	0.33	0.63
> 75	0.18	0.04	< 0.001	0.12	0.27

**Fig. 4:**
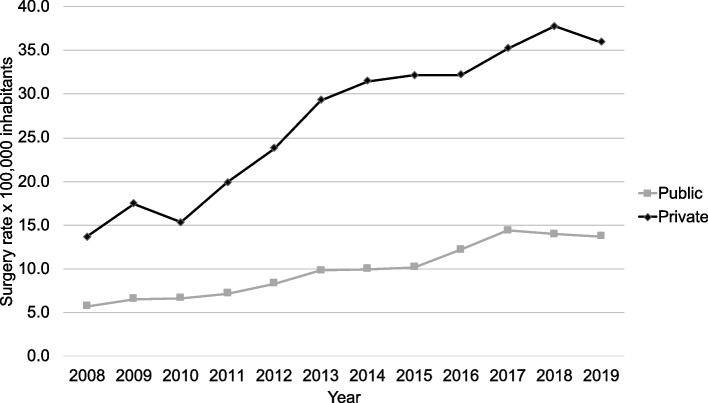
Surgery rate by 100,000 inhabitants comparing the public and the private insurance systems

When comparing based on health insurance type, 12,260 surgeries were performed through FONASA (public insurance), and 7,951 through ISAPRE (private insurances). The surgical rate for clavicular injuries in the public system was 11.1 × 100,000 and 30.9 × 100,000 in the private system, which represents a difference of 2.8 times between those healthcare systems. (Fig. 4 -Table 2). The greatest differences between these two insurance options were observed in the years 2014 and 2015 (Table 3).

**Table 3:** Surgery rate between 2005 and 2019 according to health insurance. The rate was calculated after the year 2008 because the data for the population affiliated with the public and private health insurance before that year was not available online

Year	Public insurance rate × 100,000	Private insurance rate × 100,000	Private rate / Public rate
2008	5,7	13,7	2,4
2009	6,6	17,5	2,7
2010	6,6	15,3	2,3
2011	7,2	19,9	2,8
2012	8,3	23,8	2,9
2013	9,8	29,3	3,0
2014	10,0	31,4	3,2
2015	10,2	32,1	3,2
2016	12,2	32,2	2,6
2017	14,4	35,2	2,5
2018	13,9	37,8	2,7
2019	13,7	35,9	2,6

## Discussion

The aim of this study was to describe the rate of clavicle surgeries, including clavicle fracture and dislocation, in the last 15 years and to analyze the possible effect of age, sex, and health insurance in those rates.

The main finding of this study consists on the increase of surgical rates for clavicular injuries in Chile. This is concordant with the literature published internationally, being this the first national report on clavicle surgery rates to our knowledge.

Over the 15-year study duration, the calculated surgical rate for clavicle procedures was 12.7 per 100,000 Chilean individuals aged 18 and above. Notably, the rate of clavicle fracture surgeries nearly doubled that of acromioclavicular dislocation surgeries during this period (8.03 vs. 4.69), displaying a statistically significant higher rate. Adjusting by year, a mean annual increase of 15% was observed in surgery rates (95%CI 11%-19%). The rate rose from 2.8 in 2005 to 19.1 in 2019, accounting for 10.2 times (95%CI 8.6–12.1) increase over that period of time. The reasons for the increased surgical activity are unknown.

The literature demonstrates an increase in surgical rates for clavicle fractures across multiple studies. However, research on acromioclavicular dislocation epidemiology is limited. Putnam et al., using US claims data from the IBM MarketScan Research Databases—which cover enrollment, demographic, and medical claims from over 300 large employers and 25 health plans—reported that among 95,243 patients with a clavicle fracture, 15.2% received surgical treatment. In contrast, among 52,100 patients with acromioclavicular dislocation, 5.3% received surgical treatment, representing roughly a third of the clavicle fracture rate [8]. Huttunen et al. reported in Finland an increase in the incidence of surgical treatment nearly ninefold from 1.3 per 100,000 person years in 1987 to 10.8 per 100,000 person years in 2010 [9]. Campbell et al. [10] reveals that the incidence and rate of surgical intervention for clavicle fractures has increased in Australia over the past two decades, in both sexes and across all age groups. A total of 17,089 surgical procedures were performed in Australia between the years 2001 and 2020 for the management of clavicle fractures in patients over the age of 15. The incidence of surgically treated clavicle fractures increased from 1.87 per 100,000 population, to a peak of 6.63 per 100,000 in 2016. Huttunen et al. reported that although the incidence of clavicle fractures increased in Sweden between 2001 and 2012, the rate of surgical treatment of clavicle fractures increased much more than would be expected [11]. Congiusta et al. [6] conducted a retrospective study of the US Nationwide Inpatient Sample database from 2001 to 2013 to assess operative clavicle fracture fixation. The overall proportion of patients treated with open fixation increased from 3,911 per 100,000 discharges in 2001 to 11,708 per 100,000 discharges in 2013 (2.99-fold). These results were reproduced by Schairer et al. [12] using multicenter data from patients with clavicle fractures in the US states of California and Florida, which showed an increase in the operative fixation rate from 3.7% to 11.1% between 2005 and 2010.

Sepehri et al. [7] found a significant change in surgeon practice in Canada. More clavicle fractures were treated operatively from 2007 onward: 6.9% compared with 2.2% prior to 2007. They hypothesize that this may have happened following the publication of a randomized clinical trial that demonstrated better functional outcomes and a lower proportion of malunion or nonunion following operative, compared with nonoperative treatment for midshaft clavicle fractures [4]. Schneider et al. [13] compared the pre- and post-publication proportions of displaced midshaft clavicle fractures in patients aged 16 to 60 years that were treated with surgery in two North American Level 1 trauma centers and found an increase on surgical rates from 3.7% to 34.1% of all clavicular fractures. The disparities in fixation magnitude compared with our findings can be explained by differences in the patient populations, as Schneider et al. only included younger patients with displaced midshaft fractures and such patients are more likely to have multiple orthopedic injuries, which are a relative indication for operative clavicle fixation. In our results, we observed a difference in the increase of the rate of surgeries over time that might also be influenced by the effect of scientific publications, especially those with a high level of evidence. There might be other factors influencing the observed increase in surgical rates, such as improved private insurances, greater availability of osteosynthesis devices, better-trained surgeons, and easier transmission of surgical knowledge over time. Moreover, there is a lack of consensus on the surgical management of acromioclavicular dislocation at both the national and international levels [14–16] and this may explain the milder increase observed in surgical rates for this group of patients.

Clavicle surgery rates present statistically significant differences according to the variables analyzed. In Chile, there is a higher surgical rate observed in men throughout the observation period, as well as in other countries. The male to female ratio is 1.05:1 in the US [6] 1,78:1 in Finland [9]; 4.55:1 in Australia [10] and 4.5:1 in Belgium [17]. Also, there are similarities in the age range, with the highest rate of clavicle surgery in ages between 20 and 35 years. Herteleer et al. [17] reported that the mean age of surgically treated men was 30 years old and the mean age of surgically treated women was 43 years during the period between 2006 and 2015. They found that the age-adjusted rate of surgical treatment had the inverse shape of the age-adjusted fracture incidence graph; the overall increasing rate in surgical treatment was mainly accounted for by an increasing rate of surgically treated patients who were between 20 and 70 years old. Campbell et al. [10] reported that the age group between 15 and 24 underwent the greatest number of clavicle fixations and an increased annual rate of operative intervention for all age groups. Nonetheless, the greatest rate of annual increase was noted in the group aged over 65, whilst the lowest change was noted in the 25–34 age group. Interestingly, Congiusta et al. [6] reported that the highest incidence rates were observed in the oldest age groups. Our results show that the greatest increase in the surgery rate occurred among individuals aged 25 to 34. When analyzed by diagnosis, the 15–24 age group showed a higher increase in surgeries for clavicle fractures, while the 25–34 age group had a greater increase in surgeries for acromioclavicular dislocations.

Regarding the incidence of clavicular surgery and its relationship with socioeconomic factors, such as associated health insurance, the literature is scarce. Congiusta et al. [6] reported that patients with no insurance were the least likely to undergo surgery (OR 0.63), followed by patients with Medicaid (OR 0.73) and patients with Medicare (OR 0.74). Also, patients with Medicare or Medicaid had a lower likelihood of undergoing surgery than did patients with private insurance but were not statistically difference. In our study, patients with public insurance had a lower likelihood of undergoing surgery than those with a private one. The rate in the private system triples the rate in the public health system for clavicle surgery. This difference is possibly related to the private patient’s ability to access to surgery compared with the public health insurance patients. The chilean healthcare system is significantly affected by economical segmentation with the minority of the population covered by private health insurance [18]. The difference in surgical rates might also be explained by an over indication of surgery in the private system. Furthermore, patients with private insurances are more likely to have better health literacy in contrast to persons not having a private health insurance [19]. The lower rates of clavicle surgery in the public sector could be explained by its shortage in coverage in terms of number of health care professionals per patient and concordantly the long waiting lists associated to this fact. Moreover, seek care in the private system without a proper coverage comes with a high out of pocket cost which limits public to private transfer of patients in Chile. Sepehri et al. [7] reported that the “moderate-high” quintiles [3–5] in relation to income level, had 1.18 times more chance of having surgical management than the lowest quintiles [1, 2].

As previously mentioned, surgical rate increases were greater in the male gender, the age group between 20 and 35 years, and patients with a private health insurance. However, these are associations and not necessarily causal factors. There is likely a combination of factors such as a more active population engaged in sports, greater accessibility to advanced imaging studies, continuous refinement of surgical techniques, advancements in surgical implant technology, changes in the management of chest trauma, an increase on the number of trained surgeons, and a substantial increase in both the quality and quantity of evidence supporting the benefits of surgical treatments. All these factors might synergistically explain the observed increase. Nevertheless, the lack of standardized criteria at a national (and international) level to indicate clavicle surgery, a lack of national clinical guidelines and specialized programs for the resolution of this pathology may also contribute to favor the surgical treatment of this pathology. The data extracted from the databases primarily focuses on documenting the execution of the surgical procedure, lacking in-depth information regarding the pathology details such as location, displacement, shortening, comminution, type of surgical procedure, implant use, clinical results, and the rationale behind the surgical indication.

One of the main strengths of this study is the use of a large sample size based on a nationwide database of public information that must be compulsorily recorded in all surgeries of public and private institutions. Because the information is collected from all regions of Chile, the data are expected to be representative of the country. To our knowledge, this is the first study in Chile that reports clavicle surgeries rates and analyzes its associated factors and differences with previously reported data from other countries.

One of the main limitations is the retrospective design, which can influence the recording and coding of the information. By including three different codes, it is expected to cover the greatest number of accurate diagnoses. However, we recognize that subjects may be excluded or incorrectly included and that we are unable to evaluate such potential inaccuracies inherent to diagnosis coding. Nonetheless, we believe that our findings reflect the trends in clavicle-related surgeries in Chile and may provide valuable insights for analyzing similar tends in our region.

## Conclusions

There has been a significant increase in clavicle and acromioclavicular dislocation surgeries in Chile, with disparities influenced by age, gender, and type of health insurance. Future studies may elucidate the associated factors that explain these differences and their trend over time. Our findings are in concordance with broader international trends which highlights the need for standardized clinical guidelines to more objectively evaluate surgical trends and their real impact on health.

## Data Availability

The data used are available in a public database at https://deis.minsal.cl/

## Data availability

No datasets were generated or analysed during the current study.
